# Assessing Musical Abilities Objectively: Construction and Validation of the Profile of Music Perception Skills

**DOI:** 10.1371/journal.pone.0052508

**Published:** 2012-12-28

**Authors:** Lily N. C. Law, Marcel Zentner

**Affiliations:** 1 Department of Psychology, University of York, York, United Kingdom; UNLV, United States of America

## Abstract

A common approach for determining musical competence is to rely on information about individuals’ extent of musical training, but relying on musicianship status fails to identify musically untrained individuals with musical skill, as well as those who, despite extensive musical training, may not be as skilled. To counteract this limitation, we developed a new test battery (Profile of Music Perception Skills; PROMS) that measures perceptual musical skills across multiple domains: tonal (melody, pitch), qualitative (timbre, tuning), temporal (rhythm, rhythm-to-melody, accent, tempo), and dynamic (loudness). The PROMS has satisfactory psychometric properties for the composite score (internal consistency and test-retest *r*>.85) and fair to good coefficients for the individual subtests (.56 to.85). Convergent validity was established with the relevant dimensions of Gordon’s Advanced Measures of Music Audiation and Musical Aptitude Profile (melody, rhythm, tempo), the Musical Ear Test (rhythm), and sample instrumental sounds (timbre). Criterion validity was evidenced by consistently sizeable and significant relationships between test performance and external musical proficiency indicators in all three studies (.38 to.62, *p*<.05 to *p*<.01). An absence of correlations between test scores and a nonmusical auditory discrimination task supports the battery’s discriminant validity (−.05, *ns*). The interrelationships among the various subtests could be accounted for by two higher order factors, sequential and sensory music processing. A brief version of the full PROMS is introduced as a time-efficient approximation of the full version of the battery.

## Introduction

**Figure 1:**
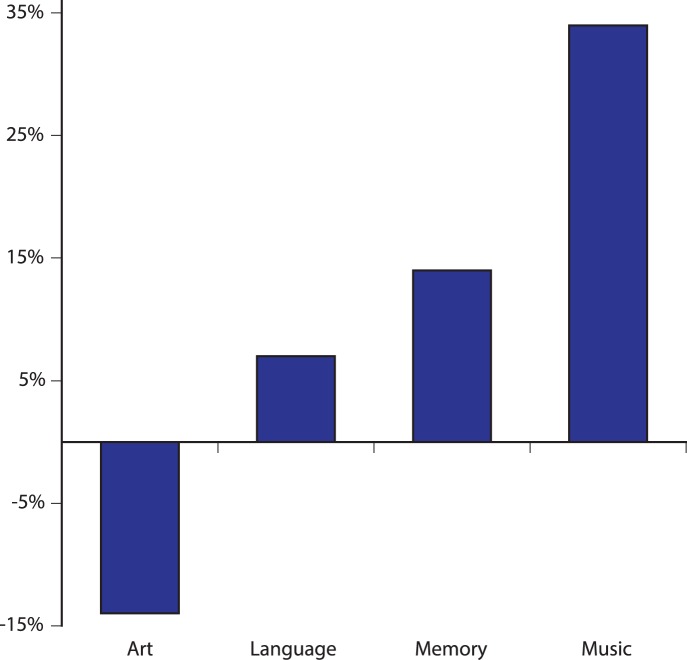
Percentage increase in publications from 2005 to 2011. Source is Web of Science. Number of publications across the time period is higher for language (539 to 817) and memory (1,140 to 2,031) than for music (81 to 162), but growth is faster in the music domain. As shown by the decrease in art-related publications, the increase in music publication is not due to a general increase in scientific publications relating to the arts.

Across sciences, interest in music has been rising steeply in recent years (see Figure 1). One reason for this development is a growing concern to understand the role of musical ability in nonmusical faculties, ranging from motor skills and general intelligence to language processing and socio-emotional competencies, such as empathy. Understanding these links might also be relevant to the understanding of deficits in these domains. For example, rhythm skills have been found to be impaired in dyslexic children and training those skills holds promise as a remedy [1]. Another reason lies in the still poorly understood origins of human musicality in terms of both its evolutionary origin and its genetic transmission [2]. Unfortunately, progress in understanding these relationships is hampered by the lack of an objective and standardized instrument to measure musical abilities. Although aspects of music perception and production have been extensively investigated [3], there has been little interest in the development of a psychometrically sound and construct-validated test capable of diagnosing individual differences in musical ability. The goal of the current research is to fill this gap.

### Current Assessment Practices

In the absence of objective measurement tools, researchers often use self-reported musicianship to estimate the presence of musical ability. In the majority of medical, neuroimaging, and psychological studies, a binary classification is used that compares the performance of musicians versus nonmusicians on variables such as general IQ and mental abilities [4], [5], brain structure [6], language processing [7], [8], vocal emotion recognition [9], memory [10], motor skills [11], and even creativity [12], to cite some recent examples.

This practice is sensible, but has a number of limitations. First, being a “nonmusician” does not, in and of itself, denote an absence of musical ability. The ability may be undiscovered, or circumstances may have prevented its development. Among the musically untrained, some people might reach a high level of musical proficiency if given the time and opportunity to do so. We refer to these individuals as *musical sleepers* because of their existing, but dormant musical skills. *Sleeping musicians*, in turn, are individuals whose musical proficiency languishes despite multiple years of training, degrees, and certificates. This metaphor, though a simplification, is useful in pinpointing the need for a tool that is capable of reducing errors of categorization by identifying individuals who have higher (or lower) musical skill than would be expected from their extent of musical training.

Second, degrees and qualifications provide at best an estimate of generic musical accomplishment. Yet, once a link between general musical ability and another ability, trait, or disorder is established, the next obvious question concerns the *type* of musical capacity that plays a key role in the relationship (e.g., tempo, pitch, rhythm, timbre, melody perception, or any combination of these). Such specific information is not only key to the scientific analysis of the relationship under examination, but could also have a role in devising treatment plans. Third, most experimental research on musical behavior today looks at neurobiological or psychological outcome variables that are measured with sophisticated instrumentation and are scaled continuously. Relating these fine-grained measures to a dichotomous predictor of uncertain validity is wasteful and bound to weaken the results.

### The Concept of Musical Ability

There is no agreement on how musical ability might be best measured with objective tasks. This is in part a result of the complexities involved in defining ability and, to an even greater extent, music. Although some of us will think of Beethoven symphonies, The Beatles’ songs, or current forms of popular music as epitomes of “music,” these are selective exemplars of an almost endless spectrum of musical varieties, as ethnomusicologists will readily point out. An important insight provided by ethnomusicological studies is the heterogeneity of music across different societies, a heterogeneity that has come to cast the Western conception of music in a new light (e.g., [13], [14]). For example, functional harmony, which relies on the sophisticated use of diatonic key relationships, has been a hallmark of much Western tonal music between 1600 and 1900, but it plays a negligible role in Indian classical music, Central African drumming music, or in much modern Western art music. Musical systems and styles also vary considerably in the emphasis they put on rhythmic organization and in the type of preferred rhythmic grouping or meter [15]. Thus, a fundamental question in developing tasks for assessing musical ability is whether the tasks are supposed to test the comprehension of a specific, culturally evolved musical system, or the ease of processing of elementary patterns of rhythm and sound that can be found across various musical systems and traditions. Our aim here was to devise a test that prioritizes the latter. However, this is a matter of emphasis and should not be equated with the goal of developing a culture-free music test.

The concept of ability similarly encompasses a variety of meanings and definitions, ranging from an understanding of exceptional ability as a result of enhancement of cognitive and physiological adaptation brought about by extended deliberate practice [16], to environmental and intrapersonal catalysts [17], to the notion of innate giftedness [18]. Our understanding of musical ability is consistent with the notion of “potential for learning music” *before* formal training and achievement ([19], p. 627). Prima facie support for the distinction between musical potential and musical training comes from the common observation that individuals with the same degree of musical acculturation appear to differ in their musical capacities, such as in the ease or speed with which they are able to reproduce a song or learn a musical instrument.

### Previous Musical Aptitude Test Batteries

Several authors around the middle of the last century developed musical aptitude batteries. Some of the more prominent of these musical aptitude tests are described in Table 1. These tests are generally very difficult to access today and were characterized as obsolete over a decade ago (e.g., [20], [21]). Some limitations of these tests stem from their objective to measure children’s generic musical aptitude (e.g., [22]–[26]). Against this background, it is understandable that the authors paid relatively little attention to the minutiae of stimulus design and control, or to the systematic revision of subtests based on item analysis and improvement.

**Table 1:** Overview of previous musical ability tests.

Test	Sample	Reliability	Validity	Format
Seashore et al.[25]	Ages 10 to 16	Internal consistency:.55 to.84 (Kuder-RichardsonFormula 21); test-retest: not reported	Convergent: yes; criterion:yes; predictive: not reported	Single 33 1/3 rpm long-playing recording
Wing [26]	Ages 8 to15	Internal consistency:.91 (split-half); test-retest:.76–.88	Convergent: yes; criterion:yes; predictive: not reported	MP3 (Italian adaption by Olivetti Belardinelli [78])
Bentley [22]	Ages 9 to 11	Internal consistency: not reported; test-retest:.84	Convergent: yes; criterion:yes; predictive: not reported	Ten-inch 33 1/3rpm disc record
E. E. Gordon [60]	Ages 9 to 18	Internal:.66–.95 (split-half); test-retest:.77	Convergent: yes; criterion:yes; predictive: yes	Compact disc
E. E. Gordon [59]	Ages 17 to 19	Internal:.83–.86 (split-half); test-retest:.79–.84	Convergent: not reported;criterion: yes; predictive:yes	Compact disc
Karma [27]	Ages 10 to 18	Internal:.68 (Kuder-Richardson); test-retest: not reported	Convergent: not reported;criterion: yes; predictive:not reported	MP3
Wallentin et al.[31]	Adult population	Internal:.69–.85 (Cronbach’s alpha); test-retest:not reported	Convergent: yes; criterion:yes; predictive: not reported	WAV and MP3

*Note.* “Not reported” means that the relevant coefficients have not been reported in the original test manual and/or in subsequent publications in current professional journals.

Specifically, one of the problems in the previous batteries was that their subtests often measured a combination of skills rather than the specific skill purportedly targeted by a given subtest. For example, in an attempt to make stimuli more “musical” than those devised by Seashore, Wing’s “rhythmic accent test” [26] and Gordon’s tempo test [23] are presented in melodic form, although the particular perceptual modalities they assess relate to timing rather than melodic skills. This makes it difficult to unambiguously attribute performances to one skill rather than to a combination of skills [27]. Another confound resulted from the use of human performers in the recording of the auditory test materials, which led to stimuli with undesirable inconsistencies in timing, timbre, and intensity between standard and comparison trials, or even slips in the performances.

A third problem is that, to the contemporary ear, many of the audio sample sounds used in previous tests sound impure or distorted, either due to limitations in recording techniques of the time, or to the quality of the audio material having degraded over time. There were also problems in the overall design of the batteries due to an unequal number or duration of stimuli within a subtest, to variations in the answer format across subtests (e.g., [22], [25], [28]), or to insufficient control of response bias and guessing patterns, which are today commonly addressed by coefficients such as *d′.*

Fourth, the procedures used for inferring test validity and reliability are tenuous by contemporary standards. Reliability estimates were based on obsolete indicators of internal consistency; test-retest reliability was examined only occasionally (see Table 1); and, with the exception of Gordon’s batteries, the validation procedures were not described in sufficient detail to allow robust inferences about the tests’ actual validity [20]. Fifth, crucial aspects of music perception skills relating to timbre, tuning, or tempo, are not assessed with the batteries currently available (see Table 2). For these reasons, it is not surprising that music aptitude batteries developed in the last century are not used in current research on music and the mind.

**Table 2:** PROMS tasks included and not included in previous music aptitude batteries.

	Previous test batteries					
PROMS Subtests	Seashore et al. [25]	Wing [26]	Bentley [22]	Karma [24]	Gordon [60], [79]	Wallentin et al. [31]
Melody	**X**	x	x	–	**X**	**X**
Rhythm	**X**	–	x	(X)	**X**	**X**
Pitch	**X**	x	x	–	–	–
Loudness	**X**	x	–	–	–	–
Accent	–	x	–	(X)	–	–
Tempo	–	–	–	–	x	–
Timbre	x	–	–	–	–	–
Tuning	–	–	–	–	–	–
Rhythm-to-melody	–	–	–	–	–	–

*Note.* **X** = musical ability subtests included in previous test batteries. x = musical ability subtests included in previous test batteries, but suboptimal (see the main text for details). (X) = subtests that are related but cannot be directly compared with each other (Karma [24] only).

Although more recent music-related test batteries are based on sound principles of test construction and validation, these batteries were specifically devised to capture deficits rather than individual differences in musical perception skills within the normal range. For example, the Montreal Battery Evaluation of Amusia (MBEA) was developed to assess amusia [29]. Another battery, the Clinical Assessment of Music Perception (CAMP), was developed to evaluate the music perception of adults with cochlear implants [30]. The Musical Ear Test (MET), exclusively measures skills in melody and rhythm perception [31]. The Goldsmith Musical Sophistication Index is a more elaborate tool to measure musical skills in the normal population. However, findings are preliminary and incomplete [32]. It is perhaps for this reason that investigators prefer to create their own tasks (e.g., [33], [34]), but these tasks do not lend themselves easily to comparisons across studies, thereby preventing the incremental accumulation of knowledge that is vital to progress in any branch of science.

### Construction of the Profile of Music Perception Skills

In order to fill the current gap in musical ability tests for normal or general adult populations, we aimed at creating a battery that should meet four criteria: (1) The test should be equally suitable for listeners who differ in the extent and in the type of their musical background; (2) the test should be more inclusive than previous batteries with respect to the musical perceptual components tested; (3) the test should assess each perceptual component with the greatest possible specificity; and (4) the test should meet contemporary standards for test construction in terms of validity and reliability. These goals made it necessary to confine the musical material to relatively basic sound patterns varying in pitch, rhythm, and timbre. The use of basic and abstract rather than complex and contextualized musical stimuli has some advantages. For example, musical compositions almost inevitably connote a certain musical system or style, thereby conferring an advantage to listeners who are familiar with the type of music being instantiated. In contrast, proto-stimuli are stylistically neutral. Furthermore, musical compositions usually conflate several perceptual features at once, thereby undermining the specificity of a subtest. Elemental music stimuli, in contrast, can be configured so as to test one specific perceptual skill at a time, leaving others aside. Perhaps full-fledged or elaborate musical passages can be manipulated in ways that ensure a similar degree of control; yet we could not see how this may be achieved.

It is important to note that the use of basic and abstract musical stimuli does not necessarily compromise criterion and predictive validity. For example, single-letter knowledge and phoneme discrimination are among the most sensitive predictors of broader measures of linguistic proficiency, such as reading ability (e.g., [35]–[38]). Similarly, the Raven Progressive Matrices test–one of the most sensitive measures of general mental ability, including numerical ability and language proficiency (e.g., [38], [39])–consists of abstract visual patterns that do not stand out as obvious items for the measurement of general mental ability. Thus, it is not unreasonable to expect that musical stimuli of similar parsimony would be predictive of real-life musical proficiency. In the selection of musical dimensions, we prioritized those that are relatively salient across musical systems and styles over others that may be highly salient in certain types of music, but play only a negligible role in others [14], [15]. Thus, we included tasks that tap perceptual sensitivity to tempo, tuning, timbre, rhythm, pitch, and melody, the first three of which were hardly examined in previous batteries. This is surprising considering that sensitivity to these dimensions appears as early as in infancy [40], [41] and that it is of great importance in the perception of almost any type of music, notably variations in expressive intent [42]–[46]. We chose to call this battery the PRofile Of Music-Perception Skills (PROMS). The term “perception” is not used in contrast to “cognition”; it denotes that our battery examines perception of music rather than production or performance of music.

## Study 1: Characterization of the PROMS and Preliminary Results

## Materials and Methods

### Participants

A total of 78 listeners participated in Study 1. They were students and staff from the university who participated in exchange for either course credit or a cash reward of 5 pounds. Because of the length of the test, 39 participants were allocated to one part of the test (Group 1: melody, accent, timbre, tempo) and the other 39 to the second part (Group 2: rhythm, rhythm-to-melody, pitch, tuning, loudness). Listeners in Group 1 were six males and 33 females (mean age = 20 years, *SD* = 2; range 18–27). Listeners in Group 2 were seven males and 32 females (mean age = 21 years, *SD* = 3, range 19–31). Twenty of 39 listeners in Group 1 and 18 of 39 in Group 2 described themselves as either music students or amateur musicians; the others had received minimal or no music education.

### Materials

From the criteria described in the introduction, we created a battery consisting of nine subtests, tapping skills across various subdomains of pitch, rhythm, and sound quality (e.g., timbre).

#### Melody

**Figure 2:**
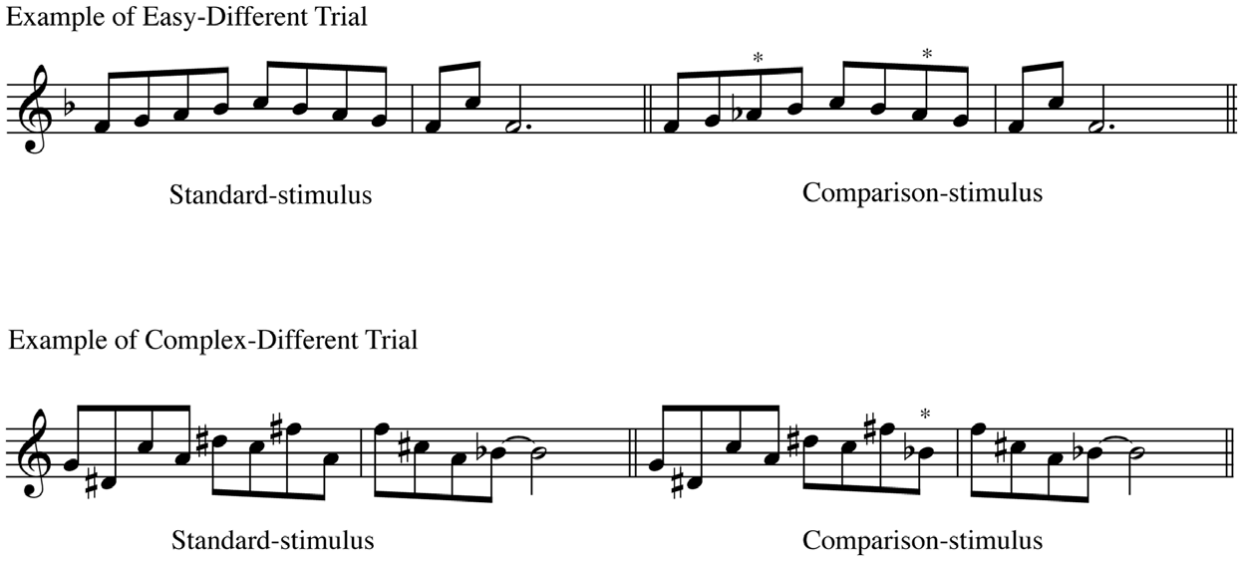
Example of melody trials. An easy trial consists of a tonal melody (upper part) as opposed to a complex trial, which is atonal (lower part). *Represents the alteration in the comparison-stimuli.

All melodies were monophonic and composed of constant rhythms (eighth notes). The musical notes of the stimuli ranged from G3 to C5 (C4 as the middle C), middle range of an 88-note keyboard/piano. The difficulty of the trials was manipulated by increasing note density and atonality. Atonal melodies are more difficult to encode compared to tonal ones (see [47], [48]). Examples of easy and complex melody structures are given in Figure 2. Stimuli were composed with the “harpsichord” timbre from Logic Pro 9 [49] because it is relatively neutral, i.e., less familiar to most listeners compared to the sound of piano, violin, or electric guitar.

#### Standard rhythm

**Figure 3:**
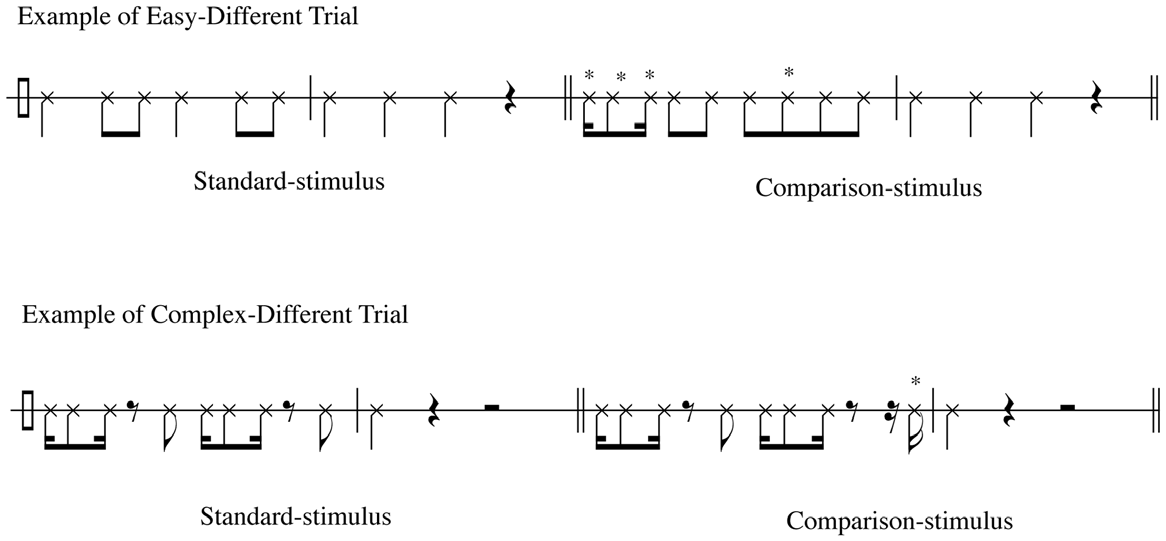
Example from the standard rhythm trials. An easy trial consists of a simple rhythm (mostly quarter notes and eighth notes), as compared with a complex trial, which consists of a more complicated rhythm (eighth notes and sixteenth notes). *Represents the alteration in the comparison-stimuli.

The standard rhythm subtest is similar to previous rhythm tests consisting of simple patterns of quarter notes, eighth notes, and sixteenth notes. The intensities of all notes were held constant. The comparison stimuli in the easy trials had one or more notes added or subtracted on the downbeat. Moderately difficult test stimuli were changed on the upbeat note. Complex trials were rhythmic patterns consisting of sixteenth notes, with test trials having rhythm alterations on sixteenth notes (see Figure 3). Each stimulus was two bars long. The rhythm subtest was delivered with “rim shot” voice from Logic Pro 9 [49] for its pure percussive, clear, and crisp timbre.

#### Rhythm-to-melody

**Figure 4:**
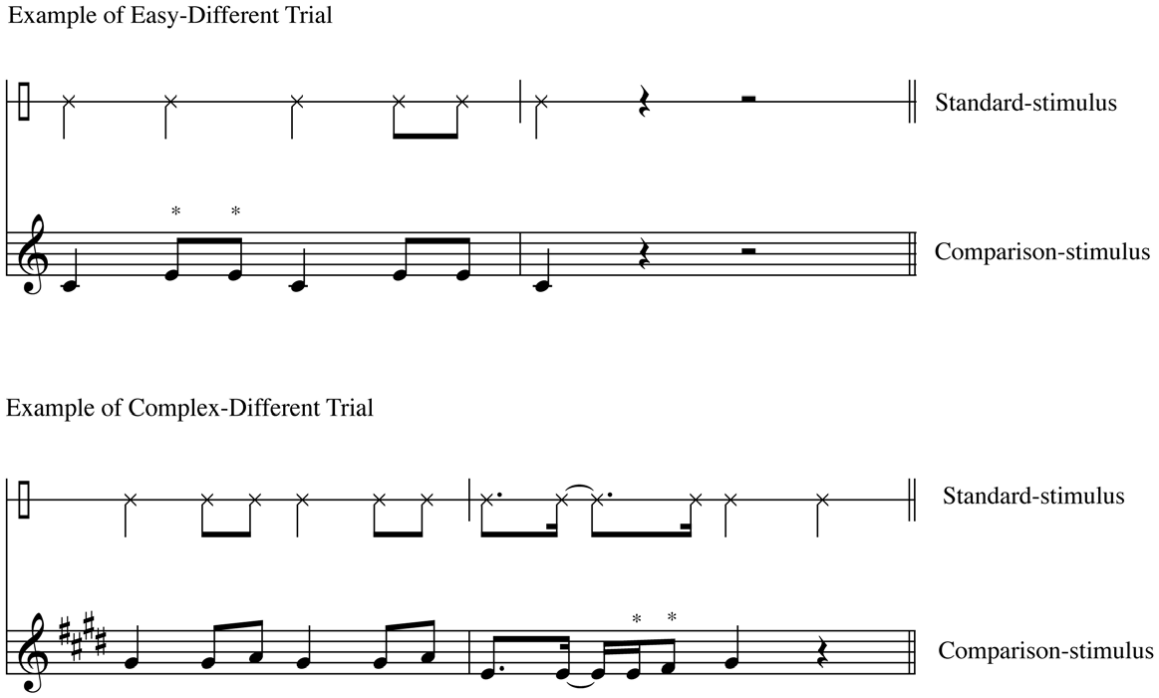
Example of rhythm-to-melody trials. An easy trial consists of a simple rhythm (mostly quarter notes and eighth notes), as compared with a complex trial, which consists of a more complicated rhythm (eighth notes and sixteenth notes). All melodies (comparison-stimuli) are tonal. *Represents the alteration in the comparison-stimuli.

This subtest introduces a novelty relative to earlier rhythm tests by targeting listeners’ ability to recognize a rhythmic pattern when it is no longer provided in its original form (i.e., in nonpitched percussive form), but embedded in a melody. Thus, listeners must attend to the rhythmic structure of a melody without being influenced by its pitch contour. Specifically, listeners are asked whether a rhythmic pattern, presented initially in percussive sound, is the same or different in a subsequently presented melodic context. All melodies in this subtest were tonal to avoid diverting listeners’ attention from rhythm to atypical melodic features (see Figure 4).

#### Accent

**Figure 5:**
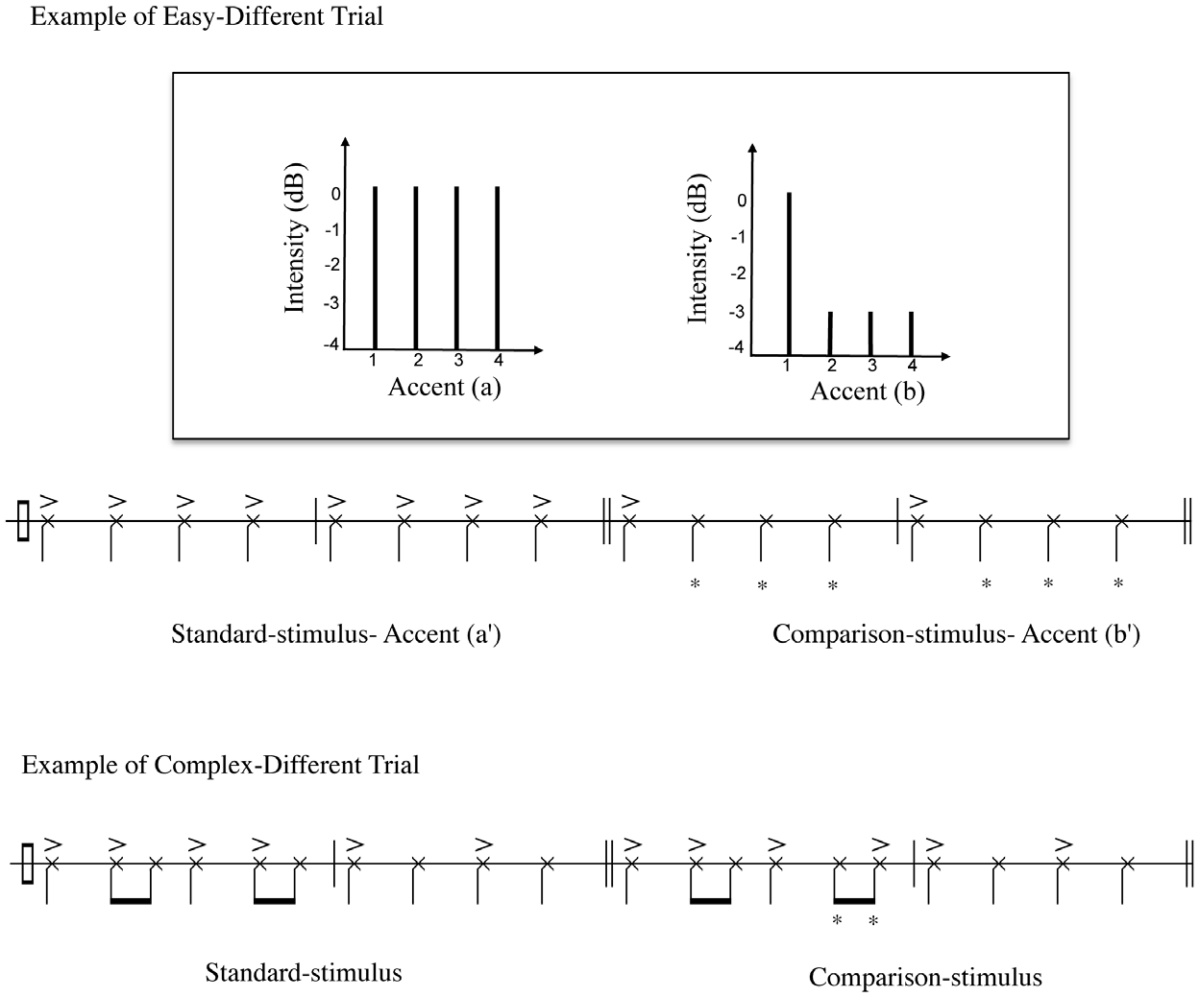
Example of accent trials. The top figure shows the level domain of the accent subtest and the bottom figure shows the time domain of the accent subtest. As the top figure shows, the intensities of the accent notes (a) are represented by the sign>in the time domain figures, Accent (a’). Accent (b) shows the unaccented notes (second, third, and fourth beats) are −3 dB lower than the accented note, which can also be seen in the comparison-stimulus in the time domain - Accent (b’). The example of a complex trial shows the alteration affecting only one or two events. *Represents the alteration in the comparison-stimuli.

This subtest assesses skills in discerning the relative emphasis given to certain notes in a rhythmic pattern. As such, it is related to the concepts of meter in music and of stress in speech. The absolute note durations (rhythms) were identical between standard and comparison stimuli. The accented notes were presented in the intensities of the other subtests, whereas the intensity of the unaccented notes was lowered by 3dB. In the easy test trials, intensity changes were applied to most sound events so as to increase the probability of detecting the alteration. In the moderate and difficult test trials, there were fewer intensity changes, which required more subtle perceptual skills to be identified (see Figure 5). As in the standard rhythm subtest, stimuli were composed with “rim shot” voice from Logic Pro 9.

#### Tempo

Listeners were presented with musical stimuli having either the same or a different tempo in the comparison stimuli. To manipulate the difficulty of the trials, the comparison stimuli differed from the standard stimuli between 7 bpm (easy) and 1 bpm (difficult). In order to attenuate the risk that preference for a given instrument or rhythm might affect the performance on this test (e.g., [50]), we used stimuli with differing rhythmic structures and timbres. The timbres were drums, bass, harmony, and melody (multilayers); conga and shaker (dual layers); and rim shot voice (monolayer). Stimuli were within the range of 110 bpm to 130 bpm, given listeners’ general preference for 120 bpm (e.g., [51]).

#### Pitch

The material of the pitch subtest was derived from a 2,000-ms sinusoid with a 250-ms linear onset and offset ramp to de-emphasize the salience of the on- and offsets [52]. The intensities of all notes were held constant. Sinusoids, or pure tones, were used in this task because use of complex tones can result in pitch changes being perceived as a result of harmonics rather than fundamental frequency [53]. The difficulty level of the pitch subtest was manipulated by varying the degree of the pitch difference between the standard and comparison stimulus (range of 7 to 50 cents, or 2 to 12 Hz, pivoting at the frequency of 440 Hz). We chose this frequency because it marks the center of a range in which music from various styles is typically composed. However, because this frequency also happens to be the standard pitch to which musical instruments are tuned for a performance (“concert pitch”), we cannot rule out that it may provide an enhanced familiarity cue to classically trained musicians.

#### Timbre

**Figure 6:**
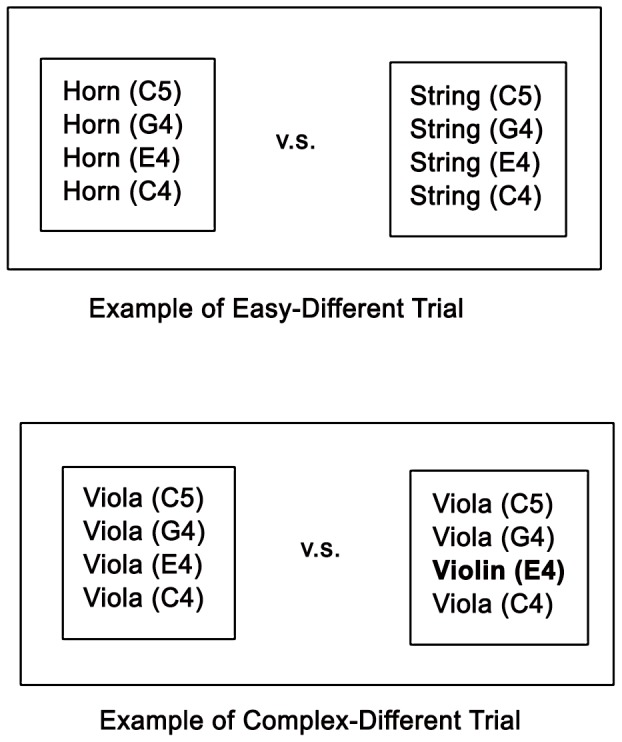
Illustration of the timbre subtest. The easy trial consists of two groups of instruments from altogether different families. In the complex trial, the instrument changes on only one note are taken from the same family (strings).

Instead of using pure tones [25], we aimed at emulating the sounds of original instruments as closely as possible. To this end, we used original instrument sounds from the Vienna Symphonic Library [54]. We used chords of four notes (C4, E4, G4, C5) to produce a rich timbre with a possibility for making very subtle changes. The duration of the chords was 1.5 s. The difficulty was varied by means of subtle changes to the instrumentation in each chord. In easy trials, the comparison was between two chords played by different families of instruments such as horn versus strings. In the moderately difficult trials, the replacement occurred in only one of the four voices (e.g., woodwind C4, woodwind E4, woodwind G4, and woodwind C5 against woodwind C4, *violin E4*, woodwind G4, and woodwind C5). In the difficult trials, the replacement occurred within the same family of instruments (e.g., a viola sound is replaced by a violin sound; Figure 6).

#### Tuning

**Figure 7:**
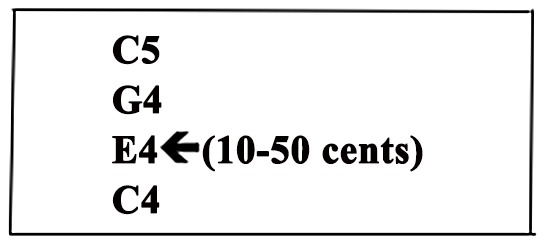
Illustration of tuning trials. The difficulty of tuning trials is manipulated by the extent to which the note E4 is shifted out of its proper frequency (from 10 to 50 cents).

As in the timbre subtest, each stimulus consisted of C4, E4, G4, and C5 to form a C chord of 1.5 s in length. This combination of diatonic harmony was chosen because it is relatively “culture free,” thereby attenuating the risk of misunderstandings about “correct tuning” due to listeners’ musical backgrounds [55]. Piano sound samples from the Vienna Symphonic Library were used. The difficulty level of the test trials was varied by subtle manipulations to the E note (a range of 10–50 cents; see Figure 7).

#### Loudness

Stimuli consisted of 2,000-ms sinusoids with a 250-ms linear onset and offset ramp to de-emphasize the salience of the on- and offsets, as in the pitch subtest. The frequency in the loudness test (440 Hz) was held constant. The intensity ranged from 3 dB higher than the standard loudness level to 6 dB below the standard level. The difficulty level of the trials was varied by the intensity difference of the standard and comparison trials: 6 to 7 dB (easy), 3 to 5 dB (moderate), and 1 to 2 dB (complex).

#### Procedure

Each subtest had 18 trials with an equal number of “same” and “different” trials. To facilitate encoding of the standard stimulus, we presented the standard stimulus twice, followed by the comparison stimulus. There was a 1.5-s interval between the standard stimulus and its repetition, followed by a 2.5-s interval preceding the onset of the comparison stimulus. We provided multiple answer options, involving levels of confidence, namely, “definitely same,” “probably same,” “probably different,” “definitely different,” and “I don’t know” (for a similar scheme, see [56]). The “probably” and “definitely” answer choices capture listeners’ confidence ratings; and the “I don’t know” option reduces guessing and response bias when the listeners are not sure about the correct response [57]. The base stimulus differed across trials to discourage listeners from relying on a fixed internal reference in their memory –a technique known as *roving* [58].

All audio files were exported to MPEG Audio Layer III (MP3) with 44.100 kHz, 128 kbps, using Steinberg Nuendo 4 in order to achieve optimal sound quality while keeping file sizes low for smooth data loading, as the sounds were delivered via a web platform (Limesurvey version 1.87). All sound samples used in this test were edited and normalized to achieve uniformity in loudness and were presented at 60 dB sound pressure level to the listeners through headphones (Audio Technica ATH-M40FS).

## Results

To calibrate the scoring to the confidence ratings, a correct response chosen with maximum confidence (“definitely same” or “definitely different”) was awarded 1 point; a correct response chosen with less confidence (“probably same” or “probably different”) was awarded 0.5 points. Incorrect responses (both probably and definitely) and the choice of “I don’t know” were awarded 0 points. After the raw score was calculated, the score was transformed to *d′* by using the standard *d′* model (z(H)–z(F)) [58]. A general interpretative framework for *d′* scores is that *d′* = 0 denotes no discrimination ability and *d′* = 1 denotes 69% correct for both same and different trials; although there is no upper limit, in general, *d′* values tend to peak around 2 [59]. By this measure, test difficulty was appropriate for the subtests of Group 2 (mean *d′* = 0.81; *SD* = 0.58), but somewhat high for the subtests of Group 1 (mean *d′* = 0.35; *SD* = 0.65).

With regards to internal consistency, Cronbach’s α for the composite score was.85 across the subtests of Group 1 and.87 across those of Group 2. Subtest coefficients ranged from a low of.48 (melody) to a high of.78 (tempo). To examine test-retest reliability, we first invited a subsample of 24 participants from Group 1 to take the test 1 week later. We used the single measure intra-class correlation with a two-way random effect model (absolute agreement definition). Test-retest was encouraging, *ICC* (22) = .82, *p*<.01 (Pearson’s *r* = .82, Spearman’s *rho = *.76; *p*<.01), prompting us to retest all 39 subjects from Group 2: *ICC* (37) = .82, *p*<.01 (Pearson *r* = .84, rho = .80; *p*<.01). For the individual subtests, retest coefficients ranged from a low of *ICC* = .56 (melody) to a high of *ICC = *.81 (timbre), both *p*’s<.01.

Test scores were also significantly related to musicianship status as defined above. In Group 1, the point biserial correlation between being a musician versus being a nonmusician (coded 1 vs 0) and the test scores was *r_pb_* (37) = .39 (*p*<.05); in Group 2, it was *r_pb_* (37) = .47 (*p*<.01). These coefficients provide initial evidence for the test’s validity, especially considering that both correlations relate to only one part of the battery.

## Study 2: Validation of the PROMS

This study was undertaken to examine improvements in the psychometric properties resulting from some revisions to the trials and to examine the test’s validity in more detail. In the case of a test of musical abilities, validation is particularly complex and daunting because of the lack of a gold standard test against which a new test may be measured. Our goal in this study was limited to an investigation of criterion validity with external indicators of musical proficiency, and of convergent validity with relevant tasks from previous tests. Because of the relatively extensive work supporting the validity of Gordon’s Advanced Measures of Music Audiation (AMMA) and Musical Aptitude Profile (MAP) [60], [61], we used these batteries for a validation of the current melody, rhythm-to-melody, and tempo test as specified below. Because Gordon’s AMMA rhythm perception task is embedded in a melodic context, we used the rhythm subtest of the MET to validate the standard rhythm subtest.

Unfortunately, there are no established tests to validate the tuning and timbre subtests. Thus, we examined content rather than convergent validity. To this end, we compiled timbre trials of a different nature (monophonic rather than polyphonic) and compared performance on the timbre subtest with performance on this newly created set of timbre stimuli. In addition, no established music tests could have been used to validate our accent, tuning, pitch, and loudness subtests. The PROMS accent subtest, however, is similar to the MET’s rhythm test, which uses percussive sound. It is therefore reasonable to also expect a positive correlation between the accent subtest and the MET’s rhythm test.

## Materials and Methods

### Listeners

Participants were 56 listeners (15 males; 41 females) aged 18 to 38 years (mean = 22, *SD* = 4.6). They were students and staff from the university who participated in exchange for either course credit or a cash reward. Seventeen students were music students, and another five students from other departments described themselves as semiprofessional musicians. Twenty listeners agreed to come back to a retest session 1 week later.

### Materials

The number of trials and subtests was the same as in Study 1. The stimulus material was slightly revised on the basis of (a) imbalances in subtest difficulty, and (b) a psychometric analysis of poorly performing trials identified in Study 1. Specifically, subtests that were comparatively too difficult or too easy were revised by replacing some of the most difficult (or easy) trials with trials of a more moderate level of difficulty. Furthermore, trials with unsatisfactory item-to-total correlations in each subtest were revised or replaced with new trials. This led to a revision in 23 out of the 162 trials (14.2%).

In addition, we assessed the listeners’ musical background with questions regarding their music qualifications (coded: Grade/Level 1–5 [ = 1]; Grade/Level 6–8 [ = 2]; bachelor degree [ = 3]; masters degree [ = 4]; PhD degree [ = 5]) and level of musicianship (coded: nonmusician [ = 1]; music-loving nonmusician [ = 2]; amateur musician [ = 3]; semiprofessional musician [ = 4]; professional musician [ = 5]). We also asked about years of musical training and involvement in critical listening activities (e.g., sound engineering, professional performance). We created a composite index of these variables (α = .83). Composites provide more reliable estimates compared with their individual components, thus protecting against Type II error (e.g., [62]).

### Procedure

There were three experimental sessions. In one session, listeners completed the current test battery. In a second session, they completed the validation tests. A subgroup of 20 listeners participated in a third session as a means for obtaining new test-retest data. The validation sessions had four external tests, namely, AMMA, MAP, MET, and timbre (monophonic). During this session, we also examined the listeners’ hearing ability by using the “air conduction pure tone audiometry procedure without masking” [63]. Prior to the music-listening part, listeners were asked to fill in the music background questionnaire.

## Results

### Descriptive Statistics

Because there are 18 trials in each subtest, the maximum score listeners can obtain is 18/18, the minimum 0/18. The level of chance performance with the current scoring system is 6.75 (if “I don’t know” is included as a response option, the level of chance is 5.4). After the raw score was calculated, the score was transformed to *d′* by using the standard *d′* model (z(H)-z(F)) [58], [59]. Table 3 shows descriptive statistics for the entire sample and, in parentheses, for the sample after removal of listeners that described themselves as either professional or semiprofessional musicians. It is of note that the group difference between the latter and nonmusicians on the pitch task was among those that did not reach significance, *t*(54) = 1.35, *p* = .18 (the others were loudness, tempo, and timbre; all *p*’s >0.10). This finding somewhat tempers the concern that the choice of 440 Hz as pivot for the pitch task might have conferred a major advantage to musicians (see Study 1, Materials).

**Table 3:** Descriptive summaries for PROMS subtests and composite score.

Subtest	Mean [Raw]	*SD* [Raw]	*Mean* [*d’*]	*SD* [*d’*]
Loudness	13.05 (13.01)*	2.61 (2.62)*	1.55 (1.10)	1.13 (1.09)
Tempo	12.88 (12.57)	2.40 (2.49)	1.45 (1.06)	1.02 (1.06)
Tuning	12.65 (11.69)	3.15 (3.37)	1.46 (1.32)	1.29 (1.31)
Standard rhythm	12.60 (12.00)	2.45 (2.58)	1.23 (1.02)	0.90 (0.96)
Rhythm-to-melody	12.31 (11.53)	3.15 (3.08)	1.33 (1.00)	1.28 (1.22)
Timbre	12.23 (11.92)	2.70 (3.12)	1.42 (1.34)	1.11 (1.26)
Pitch	12.21 (11.74)	2.40 (2.48)	1.37 (1.24)	0.97 (1.05)
Accent	11.28 (10.71)	2.49 (2.42)	0.80 (0.57)	0.89 (0.80)
Melody	10.40 (9.52)	2.56 (2.58)	0.51 (0.22)	0.91 (0.93)
COMPOSITE	109.60 (104.68)	17.88 (18.60)	1.02 (0.83)	0.67 (0.67)

*Note: N = *56.

* Values in parentheses relate to a subsample (*N = *36) in which professional and semiprofessional musicians were removed (see main text).

### Reliability

**Figure 8:**
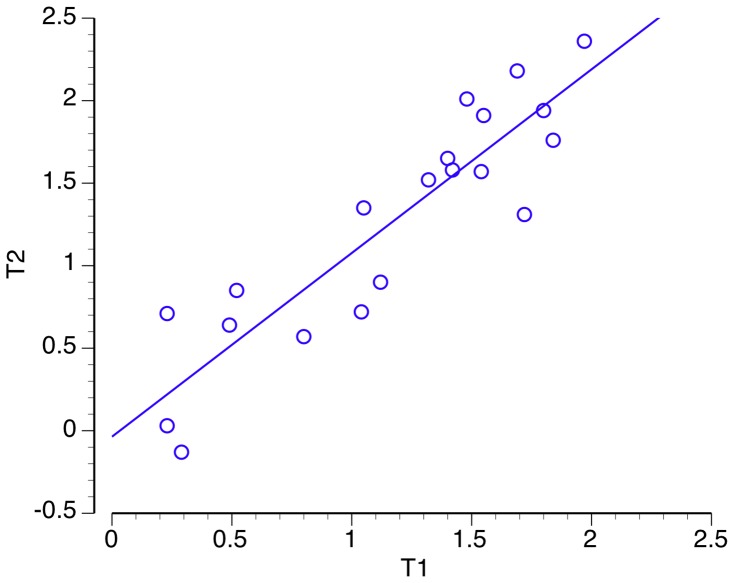
Scattergram plotting total PROMS scores at Time 1 against Time 2. Units are d prime values (*d′*).

We provide, as estimates of internal consistency, Cronbach’s α and McDonald’s ω. Omega is provided in addition to alpha because the latter is an insensitive estimate of internal consistency, notably in ability tests, where homogeneity in item content is sided with heterogeneity in item difficulty [64], [65]. As in Study 1, test-retest reliability was computed from the intraclass correlation coefficient, and based on the subsample of participants that took the test 1 week alter. Test-retest reliability for the PROMS total score was *ICC* (18) = .88, *p*<.01 (Pearson’s *r* = .90, Spearman’s *rho = *.88; both *p*’s<.01). Retest values for the individual subtests are provided in the right-hand column of Table 4. Figure 8 plots the total PROMS scores of T1 against those of T2.

**Table 4:** Cronbach’s alpha, McDonald’s omega, and test-retest coefficient for subtests and composite score.

Subtest	α	ω	Test-retest
Tuning	.81	.87	.68**
Rhythm-to-melody	.78	.83	.81**
Pitch	.73	.79	.76**
Timbre	.77	.84	.69**
Melody	.56	.73	.71**
Loudness	.72	.80	.83**
Standard rhythm	.61	.75	.63**
Accent	.55	.70	.63**
Tempo	.65	.72	.81**
COMPOSITE	.94	.95	.88**

*Note.* **p<.01. Sample size for internal consistency was *N* = 56;

test-retest was *N* = 20.

### Convergent Validity

Overall, the listeners’ performances on the current subtests were substantially intercorrelated with the tests selected for validation. Table 5 shows the validity intercorrelation matrix. In many cases, the subtests were also distinctively linked to the corresponding validation tests. Thus, the rhythm test taken from the MET correlated most strongly with both of our rhythm subtests (rhythm and rhythm-to-melody) and, not surprisingly, also showed moderate correlation with the accent subtest. Although the highest correlation of the AMMA melody was indeed with our melody subtest, it also correlated rather strongly with other test components. This could be due to the AMMA tonal test measuring more than just melodic skills, to the melodic perception skills reflecting a confluence of various musical skills, or to a combination of both.

**Table 5:** Validity correlation between AMMA, MET, MAP, and timbre (mono) with the PROMS.

Subtests (PROMS)	Tonal (AMMA)	Rhythm (AMMA)	Rhythm (MET)	Tempo (MAP)	Timbre (Mono)
Melody	**.68** **	.60**	.46**	.60**	.23
Rhythm-to-melody	.43**	**.42** **	**.64** **	.44**	.33*
Standard rhythm	.51**	.44**	**.60** **	.37**	.23
Accent	.48**	.37**	.37**	.44**	.24
Tempo	.33*	.33*	.22	**.33** *	.36**
Timbre	.30*	.27	.15	.32*	**.53** **
Tuning	.48**	.41**	.28*	.47**	**.41** **
Pitch	.34*	.33*	.12	.37**	**.49** **
Loudness	−.10	−.11	−.05	.05	.**40** **

*Note.* AMMA = Advanced Measures of Music Audiation; MET = Musical Ear Test; MAP = Musical Aptitude Profile. *N* = 52. Targeted validity correlations are in bold.

* *p*<.05.

** *p*<.01, two-tailed.

Although our tempo task was significantly correlated with the MAP tempo task, it correlated even more strongly with other test components. A likely explanation for this pattern is that the MAP tempo test heavily taxes tonal memory. It not only uses melodic sequences, but also requires listeners to judge whether the tempo of the *ending* of the melodies is the same or different compared with the ending of the standard stimulus. As such, the MAP tempo test may be a better measure of tonal memory than of tempo skills per se. To substantiate this explanation, we correlated the MAP tempo score with a *composite* score of subtests that tax memory due to their sequential nature (melody, rhythm-to-melody, standard rhythm and accent) and with a composite score of nonsequential subtests that make lesser demands on individuals’ short-term memory (tuning, pitch, loudness and timbre). As expected, the former correlation was substantially higher (*r* = .59, *p*<.01) compared to the latter (*r = *.29, *p* = .03).

### Criterion Validity

**Figure 9:**
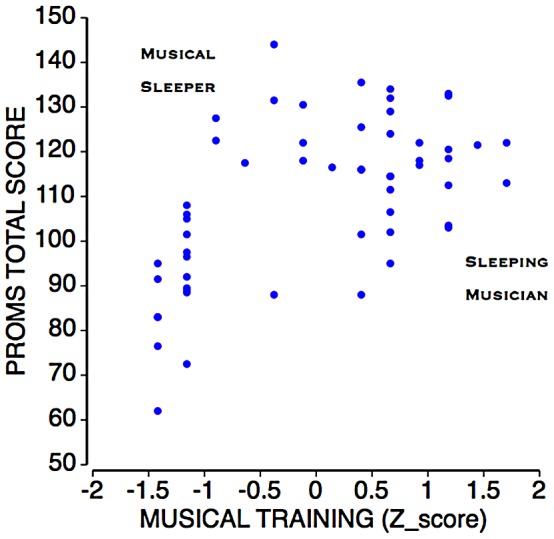
Scattergram plotting PROMS performance against an aggregate index of musical training. Training includes years of musical training, music degrees and qualifications, critical listening activities, and musicianship status (main text). Extent of training predicts PROMS performance substantially but imperfectly (*r* = .57, *p*<. 01). *Upper left corner:* Example of a “musical sleeper” performing well despite minimal musical training. *Lower right corner:* Example of a “sleeping musician” posting a lesser performance despite extensive musical training.

We found significant correlations between the total PROMS score with self-reported years of musical training, involvement in critical listening activities, music degrees and qualifications, and musicianship status of, respectively, *r*(54) = .37, *r*(54) = .45, *r*(54) = .41, and *r*(54) = .63 (all *p*’s <.01). The correlation with the composite score across the four dimensions was *r(*54) = .57, *p*<.01. This sizeable relationship between PROMS scores and indicators of external musical proficiency supports the test’s criterion validity. It is also consistent with our notion of musical sleepers and sleeping musicians, with several musically untrained participants performing well and some of the trained participants not as well (Figure 9).

## Study 3: Discriminant Validity and Test Structure

In a final study, we investigated the discriminant validity of the test battery and examined the subtest intercorrelations and factor structure underlying the nine subtests. To examine discriminant validity, we used the gap detection task with white noise. This task was chosen because it does not have a strong pitch element and it is also an established test to measure individual differences in auditory abilities [66]. Gap detection tasks were also frequently used in investigating the development of speech perception in children [67]–[69] and in hearing-impaired patients [70], [71] and have also been used to assess auditory temporal acuity and resolution (the ability to detect an auditory signal of brief duration presented at rapid rates) [68], [72], [73].

## Materials and Methods

### Listeners

Forty listeners (13 males; 27 females) aged 18 to 32 years (mean = 21, *SD* = 3.1) participated in Study 3. They were students and staff from the university who participated in exchange for either course credit or a cash reward. None of the students were music students, although six described themselves as either professional or semiprofessional musicians.

### Stimuli

The PROMS stimuli were identical to those of Study 2. We used the gap detection task as specified by Zeng and colleagues [74], who used an adaptive two-down and one-up procedure, yielding a 70.7% performance level [75]. Eleven independent 750-ms digital samples of white noise contained gaps of silence of 10 different durations at their temporal center. The gap durations ranged from 0.5 to 256 ms, in log steps, whereas the total durations remained constant. An adaptive three-alternative forced-choice procedure, with visual feedback regarding the correct response, was used to determine the gap detection thresholds. The interstimulus interval was 1,250 ms and the order of the signal and standard sounds was randomized. The test started with a medium-large signal at 32 ms to facilitate listeners’ understanding of the test. The level increased (or the difference was reduced) after two consecutive correct responses, and the level decreased after one incorrect response (two-down, one-up). If the listener made an incorrect response from two or more consecutive correct responses or vice versa, a reversal was recorded. Each run was terminated after 12 reversals or after a maximum of 70 trials. The average score from the last eight reversals was used to determine the gap detection threshold.

### Procedure

The testing procedure was the same as in the previous studies, followed by the gap detection task. The order of the sessions was counterbalanced, where half of the listeners did the PROMS first and the gap detection task second, and the other half did them in reverse order. This session lasted about 1.5 h, with a 5- to 10-min break in the middle.

## Results

### Descriptive Statistics

Gap detection scores ranged from a minimum of 1.70 ms to a maximum of 5.80 ms (*M = *2.87 ms; *SD* = .77). Descriptive statistics and psychometric properties for the PROMS were similar to those in Study 2 (Table S1). The PROMS scores were slightly lower compared to Study 2, probably a reflection of the smaller proportion of musically trained participants in Study 3 compared to those of Study 2. This probably also brought about the slight attenuation of the correlation between the test scores and the composite music education index, *r* = .38 (*p*<.05).

### Correlations between Gap Detection Task and the PROMS

Table 6 shows correlations between the gap detection task and the PROMS subtest and total scores. The lower listeners’ gap detection scores (indexed by the smaller gaps that listeners are able to detect), the better their auditory discrimination skills. Thus, a negative correlation between PROMS and gap detection scores would indicate that those who performed well on the PROMS also tended to perform well on the gap detection task. However, none of the correlations reached significance, providing support to the PROMS’ discriminant validity.

**Table 6:** Correlations between the PROMS and the gap detection task.

PROMS	Gap
Accent	−0.28
Pitch	−0.12
Rhythm-to-melody	−0.10
Melody	0.02
Timbre	0.03
Tempo	0.03
Standard rhythm	0.03
Tuning	0.10
Loudness	0.19
COMPOSITE	**−0.05**

*Note. N* = 40. All correlations non-significant.

### Factorial Structure of Test Components

Subtest intercorrelations and factorial analyses were conducted on participants from the current and the previous study combined (*N = *96). Overall, the correlations among subtests were substantial (Table 7). In analogy to Spearman’s *g*, these findings point to the presence of a generic “musicality” or *m* factor. To examine the factorial structure underlying the patterns of correlations, we ran a factor analysis with varimax rotation on the subtest scores. Two factors met the Kaiser criterion (eigenvalues >1) and were also clearly suggested by the scree plot. Melody, accent, and rhythm subtest scores were found to load highly on Factor 1. Loudness, pitch, tuning, timbre, and tempo subtest scores all loaded on Factor 2 (Table 8). From this pattern, we decided to label Factor 1 “sequential processing” and Factor 2 “sensory processing.” Of note is that the loudness subtest was only loosely connected to the other subtests. It had no cross loading on the first factor (Table 8), and comparatively modest correlations with the other subtests (Table 7).

**Table 7:** Intercorrelations of all PROMS subtest scores including the composite score.

	Comp	Tuning	Pitch	Accent	Tempo	Rhythm	R-M	Timbre	Melody
Tuning	.80**								
Pitch	.80**	.71**							
Accent	.79**	.47**	.57**						
Tempo	.74**	.60**	.59**	.50**					
Rhythm	.73**	.48**	.45**	.60**	.42**				
R-M	.69**	.49**	.44**	.55**	.39**	.61**			
Timbre	.67**	.53**	.56**	.49**	.46**	.39**	.33**		
Melody	.67**	.47**	.48**	.61**	.38**	.45**	.52**	.38**	
Loudness	.53**	.47**	.40**	.29**	.42**	.33**	.21*	.32**	.06

*Note.* Comp = composite score; R-M = rhythm-to-melody; *N* = 96.

* *p*<.05.

** *p*<.01 (two-tailed).

**Table 8:** Factor analysis of the PROMS.

Subtest	Sequential	Sensory
Melody	**.833**	.101
Rhythm-to-melody	**.781**	.191
Accent	**.757**	.348
Standard rhythm	**.690**	.330
Loudness	−.075	**.828**
Tuning	.432	**.725**
Tempo	.357	**.694**
Pitch	.477	**.682**
Timbre	.387	**.593**
Eigenvalue	4.70	1.14
% variance	52.26	12.61

*Note.* N = 96.

### Brief PROMS

Despite our efforts at keeping the test short, the full battery takes about an hour to complete. This practical disadvantage can limit its use. Thus, a brief version of the PROMS, consisting of two sensory subtests (tuning and tempo) and two sequential subtests (melody and accent), was examined on the basis of samples from the current and the previous study combined (*N = *96). This choice of subtests for the brief version was based on three considerations: First, the subtests have high loadings on their respective factors. Second, timing and pitch-related subtests are balanced with melody and tuning representing pitch tasks and with tempo and accent timing tasks. We chose the accent rather than one of the other rhythm tests because it is the more challenging of the three (see Table 3), presumably taxes grouping skills to a greater extent than do the other rhythm tests, and has an affinity with the concept of “stress” in speech, thereby lending itself to studies that compare music and speech perception. Finally, these subtests do not require the absolute silence necessary to discern subtle variations in timbre, pitch, or loudness, thereby making them more suitable to be administered online.

The brief version correlated very highly with the full version, *r*(94) = .95, *p*<.01. However, this may not be surprising, because the four brief PROMS subtests correlated with themselves in the full PROMS. We therefore examined how much variance the brief version could explain in the five subtests that were not included. To this end, we created a composite score based on excluded subtests (loudness, pitch, timbre, and the two rhythm subtests) and found that an impressive 67% of the variance in the latter could be explained by the brief PROMS. The brief version also exhibited satisfactory internal consistency (α = .84; ω = .85). Test-retest reliability, computed on the retest subsample of Study 2, was also acceptable (*ICC* = .82, *r* = .84; *rho* = .84; all *p*’s<.01). The association of the brief PROMS with the musical proficiency composite was *r*(94) = .58, *p*<.01, similar to the *r* = .57 found for the correlation between the full PROMS and musical proficiency (see also Tables S1 and S2). Although these results warrant confirmation in future studies, they attest to the promise of the brief version as a time-efficient alternative to the full version.

## Discussion

The Profile of Music Perception Skills was developed to provide researchers with an instrument to assess the level of listeners’ perceptual musicality objectively. In contrast to most other musical test batteries, which were designed for special populations (e.g., children, amusics, or adults with hearing or musical impairments), the PROMS is a test for the normal adult population. A second distinctive feature of the current battery is its multidimensionality, brought about by the inclusion of crucial, yet previously neglected, aspects of music perception such as timbre, tuning, tempo, or accent. Third, standards of test construction and validation were comparatively high. Thus, stimuli were studiously selected, balanced, and revised, and the psychometric information provided is extensive. Specifically, both internal consistency and test-retest reliability were excellent for the composite score in the three samples of Studies 1 and 2 (no retest was taken in Study 3). The reliability coefficients for the individual subtests were less impressive, but nonetheless respectable given their relatively small number of trials. Fourth, we demonstrated convergent validity with existing musical ability tests, criterion validity with external indicators of musical proficiency and discriminant validity against a purely psychoacoustic, nonmusical test. Fifth, results that could be compared across the various samples replicated well. Thus, criterion validity correlations with indicators of musical proficiency were consistently significant and sizeable across the four samples; test-retest coefficients were consistently high across the three retest-samples; and means and standard deviations were similar in Studies 2, 3 and in an ongoing Internet study (Tables S1, S2, S3).

### Uses of the Battery

The current instrument has several potential uses. First, it should help to attenuate errors of categorization that result from relying on the self-reported extent of musical training only. For instance, several participants in our study scored far better (or worse) on the test than was to be expected from their extent of musical training. This finding is consistent with the distinction of *musical sleepers* and *sleeping musicians,* that is, musically untrained but capable individuals, and, vice versa, highly trained individuals of limited musical ability. It is easy to see how routinely allocating musically skilled and unskilled nonmusicians to one single group of nonmusicians–all presumably lacking in musical skill–can lead to distorted estimates and interpretations of the effects of musical ability on any outcome, be it language processing, autism spectrum disorder, or brain anatomy. The current battery should be helpful in improving the sensitivity of musical ability assessments, especially when used in combination with musical training indicators.

Thus, when a high PROMS score is sided with advanced musical qualifications, one might infer musical proficiency with maximum confidence. Such confidence is lessened when the same qualifications are paired with a modest PROMS performance. Musically untrained individuals who score high on the PROMS, in turn, might represent a special group of musically gifted individuals, who may exhibit a very different response pattern in outcome measures compared to untrained individuals with low PROMS scores. Of course, this is only a suggestion of how multiple measures of musical capacity may be best combined for assessment purposes, and its merits must be examined in future research.

Second, categorization based on musicianship usually only allows linking an outcome to musical ability or expertise generically, but not to any specific musical skill. With the current battery, the user is able to obtain information on specific musical perception skills. Thus, any link between musical ability and a nonmusical ability–be it language processing, working memory, or vocal emotion recognition–can be understood in more detail than is currently possible. Isolating the specific musical components that underlie the relationship between musical ability and other abilities or disorders is not only important for understanding the latter, but it may also play a role in devising effective treatment plans.

Third, the PROMS may have some uses in special populations. For example, hearing aids enhance speech perception, but do little to improve the quality of music perception in hearing-impaired populations. Just why the corrective devices do so little to restore music perception is far from clear (e.g., [76]). Comparing population norms of normally hearing adults to the performance of populations with hearing impairments on standardized batteries such as the PROMS could help to particularize the type and extent of their musical deficits.

Finally, the PROMS may be useful as a tool for researching the nature of music perception itself. Music is conventionally partitioned into distinct features such as rhythm, meter, tempo, melody, harmony, timbre, and so on, but little is known about the structure of the *perception* of these various musical features. Although this issue can be addressed through experiments [77], the factorial analysis of interrelationships in distinct perceptual skills represents an important complementary strategy to experimentation. For example, one might have expected tonal and temporal processing to emerge as basic factors in music perception [78]. Yet, the current work found that interrelationships among the various subtests could be best accounted for by sequential and sensory music processing modules. It is noteworthy that both timbre and tuning trials have a component of concurrent sound segregation that is enhanced in musicians [79]. Thus, the current factor structure, more than reflecting temporal and pitch processing modules, seems more suggestive of the distinction between sequential and concurrent processing germane to work in auditory neuroscience (e.g., [80]).

### Limitations and Future Directions

Though a major step forward relative to earlier batteries, the PROMS is not a perfect or exhaustive test of musical ability. First, individual differences in the perception of higher order musical qualities such as phrasing, balance, and musical expression are not measured with the present battery, nor are musical production skills. The main reason for leaving out production tasks is that, with the possible exception of some basic motor skills such as tapping or simple finger sequencing, production tasks would confer an advantage to those with experience in handling a musical instrument, including the human voice. As such, they are likely to measure the extent of such practice rather than aptitude for musical performance. However, the current battery can be used to examine whether perceptual skills and production or performance skills are related, a question that has received little attention to date.

Second, although comparatively ample evidence for the battery’s convergent, discriminant, and criterion validity was obtained in the current studies, the validation of any test battery is a continuous process requiring studies of a different kind. As long as a gold standard test for musical aptitude does not exist, the focus of future studies ought to be on predictive and criterion validity rather than on convergent validity. For example, professional groups with known skills in a given music domain (tuning and timbre sensitivity in piano tuners, rhythmic abilities in percussionists) should perform particularly well on those subtests that relate to their musical expertise. Musical novices’ scores on the test, taken before they start musical instruction, should be moderately predictive of the ease with which the students acquire skills in understanding and/or producing music over time. Such information will be valuable, but will take years to collect.

Third, a rigorous examination of the battery required repeated and lengthy testing sessions that limited the number of people that could be tested. However, because several findings replicated across the samples and studies, they are unlikely to include distorted estimates or false positives. Even so, the results warrant confirmation in further studies, and collection of data from larger and more diverse samples is an important next step to take. The short version of the PROMS introduced in Study 3 should be helpful in the gathering of data from large and diverse samples. These data will help to address questions related to the distribution of musical skills in the general population, for example, whether distributions vary according to parameters such as age, gender, and socioeconomic status, or to the presence of strong musical institutions.

In conclusion, the absence of work on musical ability test batteries stretching over 30 years presents a hurdle to progress in the research on the neural and psychological foundations of the musical mind, including work on its relation to nonmusical processes such as language processing, emotion recognition, or motor coordination. It is hoped that the current battery can facilitate this work.

### Note

Order of authorship was determined alphabetically. Michael Cheung helped with the programming of the Gap Detection Task. Tuomas Eerola kindly provided the validation sounds for the timbre subtest.

## Supporting Information

Table S1 Overview of key results of the PROMS across studies.
(DOCX)

Table S2 Overview of key results of the Brief PROMS across studies.
(DOCX)

Table S3 Preliminary data from an ongoing Internet Study.
(DOCX)
