# Supplementary material for: Assessing Musical Abilities Objectively: Construction and Validation of the Profile of Music Perception Skills
Source: PLoS One. 2012 Dec 28;7(12):e52508. doi: 10.1371/journal.pone.0052508 (PMC3532219; doi:10.1371/journal.pone.0052508)
Supplement: Table S1 — Overview of key results of the PROMS across studies. (DOCX) [file pone.0052508.s001.docx]

**Table S1**. Overview of key results of the FULL PROMS across studies.

| **Study** | **Sample size (*N*)** | **Mean** | ***SD*** | **Mean *d’*** | ***SD* *d*’** | **α** | **ω** | **r_tt_^c^** | **Music education^d^** |
| --- | --- | --- | --- | --- | --- | --- | --- | --- | --- |
| Study 1  Group 1 ^a^ | 39 | 40.42 | 8.78 | 0.35 | 0.65 | .87 | .92 | .82** | .39** |
| Study 1  Group 2 ^b^ | 39 | 57.50 | 8.42 | 0.81 | 0.58 | .85 | .93 | .84** | .47** |
| Study 2 | 56 | 109.60 | 17.88 | 1.02 | 0.67 | .94 | .95 | .90** | .57** |
| Study 3 | 40 | 102.56 | 13.94 | 0.74 | 0.51 | .89 | .88 | NA | .38* |

*Note.* Cumulative *N* =174 (Studies 1 to 3)

**p*<.05. ***p*<.01 (two-tailed).

^a^ Group 1 (melody, accent, timbre, tempo)

^b^ Group 2 (loudness, rhythm, rhythm-to-melody, pitch, tuning).

^c^ Test-rest coefficients are computed from the *ICC*

^d^Coefficients are Pearson correlations between the PROMS total score and the composite index of music education (see Study 2), except for Study 1 (see main text).
