# Supplementary material for: Assessing Musical Abilities Objectively: Construction and Validation of the Profile of Music Perception Skills
Source: PLoS One. 2012 Dec 28;7(12):e52508. doi: 10.1371/journal.pone.0052508 (PMC3532219; doi:10.1371/journal.pone.0052508)
Supplement: Table S2 — Overview of key results of the Brief PROMS across studies. (DOCX) [file pone.0052508.s002.docx]

**Table S2.** Overview of key results of the Brief PROMS across studies.

| **Study** | **Sample size (*N*)** | **Mean** | ***SD*** | **Mean *d’*** | ***SD* *d*’** | **α** | **ω** | **r_tt_^b^** | **Music education^c^** |
| --- | --- | --- | --- | --- | --- | --- | --- | --- | --- |
| Study 1  (Group 1)^a^ | 39 | 40.42 | 8.78 | 0.35 | 0.65 | .87 | .92 | .82** | .39** |
| Study 2 | 56 | 47.21 | 8.69 | 1.05 | 0.83 | .87 | .88 | .82** | .62** |
| Study 3 | 40 | 44.14 | 7.09 | 0.72 | 0.65 | .81 | .85 | NA | .49** |

*Note. N* = 135 (Studies 1 to 3)

**p*<.05. ***p*<.01 (two-tailed).

^a^ We include the results of Group 1 because the subtests of Study 1 are the same as those later used in the Brief PROMS (melody, accent, tempo, tuning), except for tuning, which replaces the timbre subtest.

^b^ Test-rest coefficients are computed from the *ICC*

^c^ Coefficients are Pearson correlations between the PROMS total score and the composite index of music education (see Study 2), except for Study 1 (see main text).
