# Supplementary material for: Assessing Musical Abilities Objectively: Construction and Validation of the Profile of Music Perception Skills
Source: PLoS One. 2012 Dec 28;7(12):e52508. doi: 10.1371/journal.pone.0052508 (PMC3532219; doi:10.1371/journal.pone.0052508)
Supplement: Table S3 — Preliminary data from an ongoing Internet Study. (DOCX) [file pone.0052508.s003.docx]

**Table S3.** Preliminary data from an ongoing Internet Study.

| **Internet**  **Study** | **Sample size (*N*)** | **Mean** | ***SD*** | **Mean *d’*** | ***SD* *d*’** | **α** | **ω** | **r_tt_** | **Music education** |
| --- | --- | --- | --- | --- | --- | --- | --- | --- | --- |
| PROMS | 249 | 107.73 | 14.91 | 0.97 | 0.58 | .91 | .88 | NA | .35** |
| Brief PROMS | 249 | 45.96 | 7.36 | 0.97 | 0.72 | .84 | .80 | NA | .36** |

*Note.* Data collection and analysis of this ongoing study are preliminary and not completed. The results are reported here only to indicate how a larger and more diverse population might be expected to perform on the PROMS. Participants are recruited through Amazon’s Mechanical Turk [81] and complete an online version of the full-length PROMS. The current preliminary sample consisted of 52.3% female and 47.7% male participants, aged between 15 and 74 years (*M* = 29.3; *SD* = 10.4). 50.6% of participants had no college or university education. Participants came from 47 different countries, most of them from the Unites States (47%), the UK (10%) and Canada (6%).
